# Echocardiographic Evaluation of the Right Ventricular Thickness, Myocardial Visualization, and Fractional Area Change: The Impact of Contrast Agent and Transducer Selection

**DOI:** 10.1111/vru.70153

**Published:** 2026-03-05

**Authors:** Yeonju Park, Suyeon Yoon, Sumin Han, Jihye Shin, Minhyung Kim, Seungjo Park

**Affiliations:** ^1^ Department of Veterinary Medical Imaging, College of Veterinary Medicine and BK21 FOUR Program Chonnam National University Gwangju South Korea

**Keywords:** contrast‐enhanced echocardiography, myocardial delineation, myocardial thickness measurement, right ventricle segmentation

## Abstract

Accurate assessment of right ventricular wall thickness, myocardial visualization, and chamber dimensions is crucial in veterinary cardiology but remains understudied in dogs. This prospective observational study evaluated 10 healthy beagle dogs to compare three echocardiographic approaches for assessing right ventricular parameters: sector transducer, convex transducer, and convex transducer with contrast agent. The dogs’ health was confirmed through physical examination, laboratory testing, and echocardiographic examinations. Statistical analyses were performed using paired sample *t*‐tests, analysis of variance with Scheffé’s post hoc test, Kruskal–Wallis tests for multiple comparisons, and intraclass correlation coefficients (ICCs) for observer reliability, with significance set at *p* < 0.05. Using a myocardial visibility and delineation scoring system, the study demonstrated that convex transducers offer superior visualization and inter‐observer agreement, particularly in near‐field segments (ICC: 0.12–0.92 vs. 0.06–0.73). Apical visualization of the right ventricle was significantly improved by contrast‐enhanced echocardiography, thereby enhancing the reliability of right ventricular fractional area change measurements (mean RVFAC, 36.1% ± 7.3% with contrast, ICC 0.7). Additionally, myocardial‐chamber delineation was improved by contrast‐enhanced echocardiography, which highlighted its potential for evaluating specific pathological conditions, such as myocardial tumors or lesions. This study compared different echocardiographic approaches, clearly identified each technique's clinical strengths and limitations, and emphasized the utility of convex transducers and contrast agents, particularly in near‐field lesions and complex myocardial abnormalities assessment. These findings provide valuable insights into improving echocardiographic technique reliability and accuracy in veterinary cardiology and offer practical contributions to canine heart disease diagnosis and treatment planning.

**Abbreviations**
CT‐B mode: convex transducer with b‐mode
CT‐CE mode: convex transducer with contrast‐enhanced mode
ICC: intraclass correlation coefficient
LCTHB: left cranial transverse heart base
LV: left ventricle
RPFC: right parasternal four‐chamber
RV: right ventricle
RVFAC: right ventricular fractional area change
RV‐LAFC: right ventricle‐focused left apical four‐chamber
ST‐B mode: sector transducer with B‐mode

## 1 Introduction

In dogs and cats, the right ventricle (RV) is affected by several diseases, such as pulmonary hypertension, left‐sided heart disease, arrhythmogenic right ventricular cardiomyopathy, congenital heart defects, pericardial disease, valvular malformations, and tumors [1]. Therefore, in veterinary medicine, noninvasive RV assessment is crucial for accurate diagnosis and effective disease management. However, because of structural and functional factors, quantitative RV evaluation is challenging. Because RV morphology and functional indices are influenced by anatomical complexity and loading conditions, imaging optimization is required for reliable assessment [1, 2, 3]. In veterinary cardiac function assessments, the RV is relatively understudied compared with the left ventricle (LV), and echocardiographic evaluation often relies on qualitative assessment because quantitative parameters can be inaccurate [4, 5]. Although visual assessment is important for RV evaluation, quantitative methods are needed to improve accuracy and reproducibility [6].

In veterinary medicine, several imaging modalities, including thoracic radiographs, echocardiography, computed tomography, and magnetic resonance imaging (MRI), are used to assess the right heart. Cardiac MRI is considered the most accurate method for RV morphology and function evaluation in humans [7, 8, 9]. However, its use in veterinary medicine is limited by high costs, the need for anesthesia, and technical and equipment requirements. Additionally, its application in veterinary practice is constrained by the lower incidence of coronary artery disease in domestic animals and the reliability of echocardiography for the diagnosis and monitoring of common cardiac conditions in dogs and cats [10]. Therefore, because of its simplicity and comprehensive diagnostic capabilities, echocardiography remains the most widely used and versatile technique of RV assessment in both veterinary and human medicine [6].

Accurate measurements of RV wall thickness are essential for disease severity assessment and informed treatment strategies. For example, severe RV concentric hypertrophy has been associated with poor prognosis in pulmonic stenosis [11]. In humans, increased end‐diastolic thickness of the RV's free wall is reported as a predictor of pulmonary hypertension [12]. In dogs, there are reports on the RV's free wall thickness being obtained from M‐mode images in the right parasternal short‐axis view [6]. However, when the M‐mode is used, the thickness can be assessed at a single point, which limits the evaluation of the entire RV thickness. Therefore, to comprehensively assess RV thickness, multiple views providing optimal RV visualization are required.

Right ventricular fractional area change (RVFAC) is used for right ventricular systolic function assessment. Compared with other cardiac metrics, echocardiography‐measured RVFAC exhibited the strongest correlation with MRI‐derived right ventricular ejection fraction (*r* = 0.80, *p* < 0.001) [13, 14, 15]. Therefore, RVFAC serves as a surrogate marker for right ventricular ejection fraction. RVFAC is determined by tracing the RV endocardium during systole and diastole. However, because this measurement's accuracy heavily depends on imaging quality, it is impractical when the endocardium is poorly visualized [3]. Because of the difficulty in echocardiography visualization, it is challenging to accurately identify the right ventricular myocardium's border, which makes evaluation methods like FAC measurement challenging.

Ultrasound contrast agents are made of stabilizing shell‐encapsulated gas‐filled microbubbles [16]. Microbubbles generate second and third harmonic ultrasound backscatter signals distinguishable from the primary fundamental frequency responses of normal tissues [16]. Because they remain in the vasculature as blood pool markers, microbubbles highlight blood flow and tissue perfusion [17]. SonoVue (Bracco, Milan, Italy), a second‐generation contrast agent, consists of stabilized sulfur hexafluoride microbubbles, each with a mean size of 2.5 µm, encased in a phospholipid shell [18]. SonoVue, which is available for use in Asia, Europe, and North America, has been reported in veterinary medicine, particularly for liver and spleen evaluation [19, 20, 21]. However, few studies have evaluated its application in cardiac imaging. A previous study reported that using the modified Simpson method, SonoVue improves left ventricular chamber opacification and aids cardiac output measurement in dogs [22]. The use of contrast agents has been reported to improve right ventricular visualization in human patients with congenital heart disease, allowing for a more accurate RV dimension and function assessment [23]. However, studies on the application of ultrasound contrast agents for the RV are limited, suggesting potential benefits in enhancing RV visualization and improving functional assessment accuracy.

Sector transducers are traditionally used for echocardiography, and their small footprint and high frame rates facilitate intercostal, parasternal, and apical imaging [24]. By contrast, convex transducers provide higher frequencies and improved spatial resolution, though at lower frame rates [25]. Accordingly, transducer choice can influence RV assessment. To the best of our knowledge, the performance of convex transducers for veterinary cardiac assessment has not been systematically evaluated.

This study sought to improve right ventricular visualization, present mean values of right ventricular myocardial thickness in beagles through accurate measurement, and enhance RVFAC measurement accuracy using sector or convex transducers with contrast echocardiography. This study explored a new approach to echocardiography by comparing the visualization, thickness, and area measurement accuracy of sector and convex transducers, as well as analyzing the impact of contrast agents on RVFAC measurements. It is hypothesized that compared with sector transducers, using a convex transducer and a contrast agent can achieve superior visualization, improve myocardial thickness measurement accuracy, and increase RVFAC measurement precision.

## 2 Materials and Methods

### 2.1 Study Subjects

This study used 10 adult, purpose‐bred beagle dogs (eight intact males and two intact females; weight 7.7–11 kg, mean: 9.6 kg; age 1–5 years, mean: 3.2 years). Based on physical examination, complete blood count, serum chemistry, heartworm testing, thoracic radiographs, and echocardiography, all dogs were clinically healthy. None of the dogs had a history of treatment with drugs that affect the cardiovascular or respiratory system, and no medications that could influence cardiac dynamics, including sedatives and anesthetics, were administered during examinations. The dogs were provided with tap water ad libitum, and they were fasted for about 12 h before the experiment. Prior to echocardiographic examination, the hair over the thoracic region was clipped. Echocardiographic studies were performed over a cut‐out in the examination table, with the animals initially in right recumbency, followed by left recumbency, with simultaneous electrocardiogram recording. Pulmonary regurgitation was exhibited by one dog and tricuspid regurgitation by two dogs. Because these conditions were considered minor and unlikely to significantly affect the overall evaluation of the study, these dogs were included in the analysis. The dogs were cared for as per the Laboratory Animal Research Center Guidelines for Care and Use, and ethical approval of the experiment protocol was granted by Chonnam National University's Institutional Animal Care and Use Committee (approval number: CNU IACUC‐YB‐2024‐111).

### 2.2 Instruments

**FIGURE 1:**
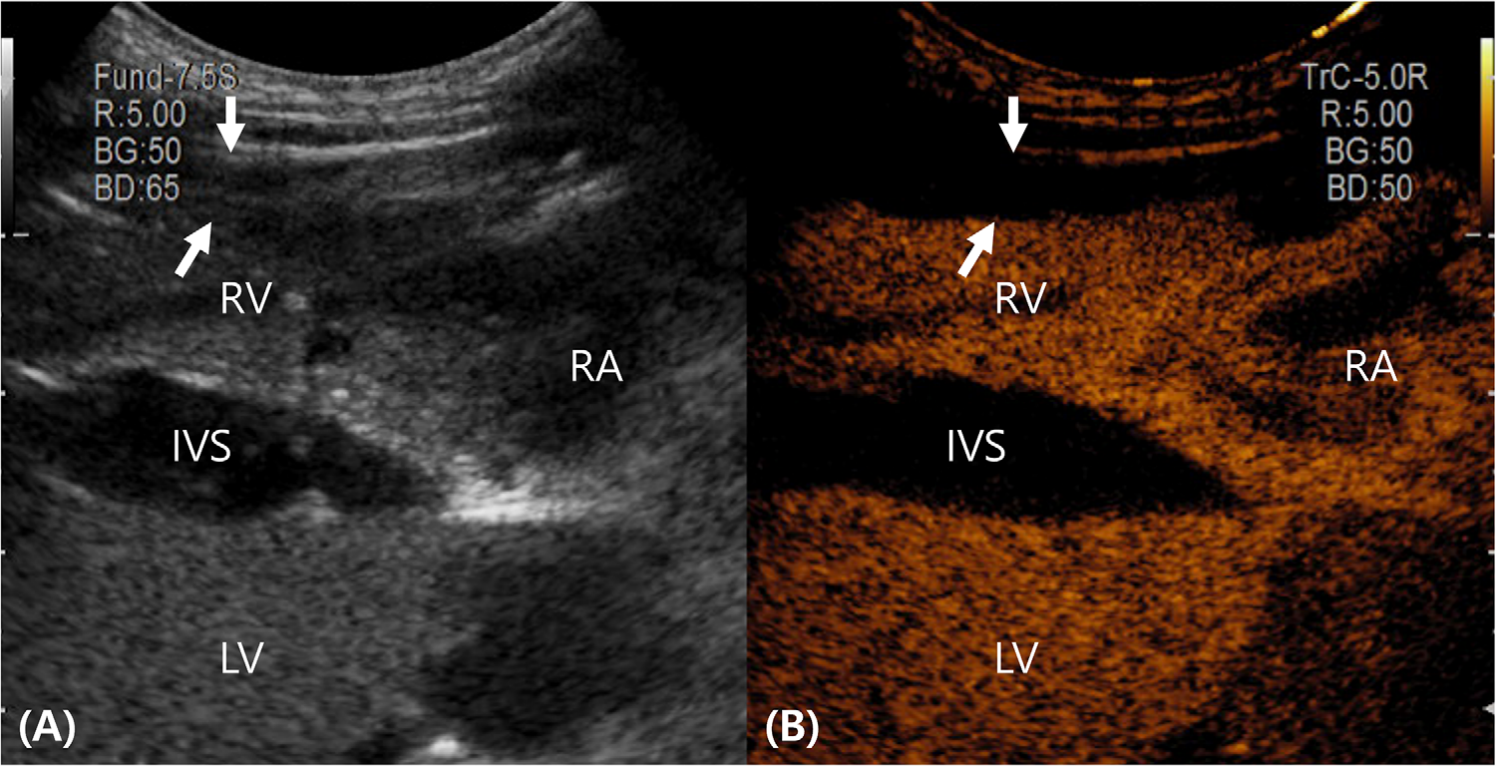
The dual ultrasound image mode during a contrast‐enhanced right parasternal four‐chamber view. The images are oriented with the left side cranial and the right side caudal, and the near field corresponds to the right ventricular free wall. Ultrasound contrast images were acquired using a convex transducer. (A) The gray scale image was reconstructed from the contrast pulse sequence. (B) The contrast image is displayed in an orange or sepia hue to visualize contrast agent distribution. The myocardium of the right ventricular free wall (arrows) and the IVS exhibit uniform mild enhancement. IVS, interventricular septum; LA, left atrium; LV, left ventricle; RA, right atrium; RV, right ventricle.

An Arietta 750 ultrasound machine (Hitachi Medical Corporation, Tokyo, Japan) with a sector transducer (S31) and convex transducer (C421) was used. The sector transducer was operated at a frequency of 2–9 MHz, with a mechanical index and frame rate of 1.23 and 117, respectively. The convex transducer was operated at a frequency of 3–12 MHz with a mechanical index and frame rate of 1.07 and 29, respectively. The convex transducer in contrast harmonic imaging mode was used to perform cardiac contrast echocardiography. For the B‐mode and contrast‐enhanced image, the mechanical index was set at 0.09 and 0.13, respectively, with a frame rate of 12. The dual image mode, whereby the left side displays a gray scale image and the right side is rendered in an orange or sepia hue to emphasize the regions enhanced by the contrast agent, was used during contrast‐enhanced ultrasound (Figure 1). To optimize RV wall visualization, the depth was set at 4–6 cm. Images and videos were stored and analyzed using a picture archiving and communication system (INFINITT Healthcare, Seoul, Korea).

### 2.3 RV Segmentation in Echocardiographic Views

**FIGURE 2:**
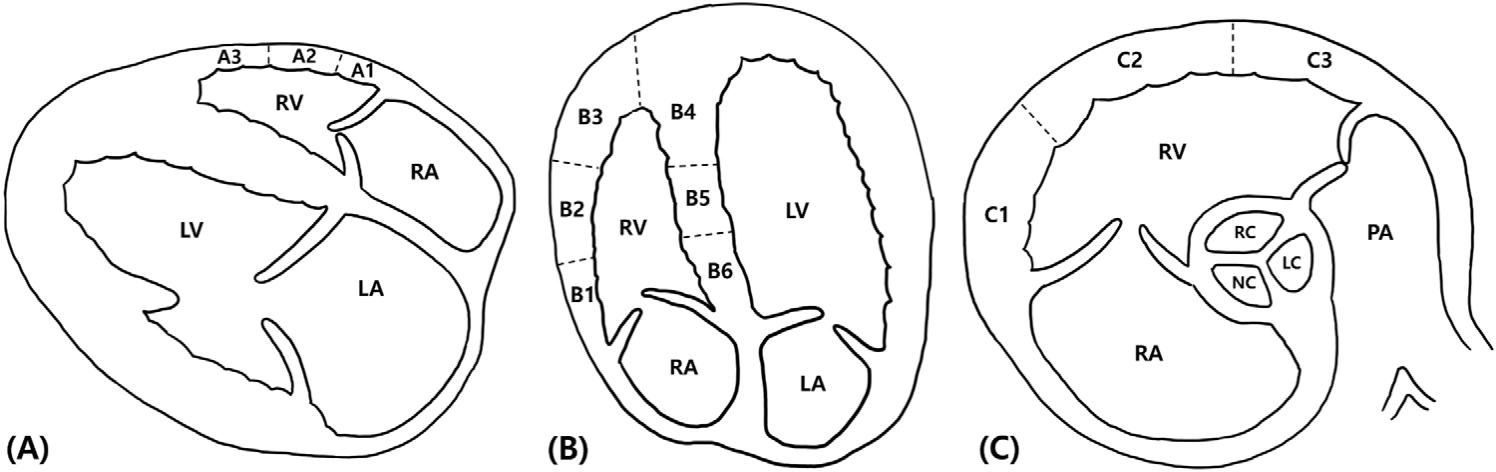
RV segmentation. (A) In the right parasternal four‐chamber view, the RV wall was divided into segments A1 (basal RV free wall), A2 (mid‐RV wall), and A3 (apical RV wall). (B) In the RV‐focused left apical four‐chamber view, the RV wall was divided into segments B1 (basal RV free wall), B2 (mid‐RV free wall), B3 and B4 (apical RV wall), B5 (mid‐septal wall), and B6 (basal septal wall). (C) In the left cranial heart base view, the RV wall was divided into segments C1 (near‐tricuspid valve wall), C2 (mid‐RV wall), and C3 (near‐pulmonary valve wall). LA, left atrium; LC, left coronary cusp of the aortic valve; LV, left ventricle; NC, noncoronary cusp of the aortic valve; PA, pulmonary artery; RA, right atrium; RC, right coronary cusp of the aortic valve; RV, right ventricle.

The RV's visualization and thickness were evaluated in the RV‐focused left apical four‐chamber view, right parasternal four‐chamber (RPFC) view, and left cranial transverse heart base (LCTHB) view. In the RPFC view, the RV's free wall was divided into segments A1 (basal RV free wall), A2 (mid‐RV wall), and A3 (apical RV wall) (Figure 2A). In the RV‐focused left apical four‐chamber view, the RV wall was divided into segments B1 (basal RV free wall), B2 (mid‐RV free wall), B3 and B4 (apical RV wall), B5 (mid‐septal wall), and B6 (basal septum wall) (Figure 2B). The LCTHB view, which corresponds to the RV base, was divided into segments C1 (near‐tricuspid valve wall), C2 (mid‐RV wall), and C3 (near‐pulmonary valve wall) (Figure 2C). Given the lack of consensus, literature‐based standards for RV segmentation in veterinary echocardiography, segmentation was performed according to predefined, view‐specific criteria to ensure consistency.

Two independent observers, Y. Park and S. Park, both specializing in veterinary diagnostic imaging, participated in the study. One observer was the most experienced individual in echocardiography and cardiac imaging among the available personnel, and the other was the lead investigator of this research. The first observer, a DVM and PhD in veterinary medical imaging, has 12 years of experience in echocardiography and cardiac imaging in dogs, whereas the second observer, holding a DVM degree, has 1.5 years of experience in the same field. Measurements and myocardial visibility scores recorded by each observer were blinded to the other observer. The observers measured each segment three times during systole and diastole. Myocardial thickness was measured at end‐diastole (the onset of the QRS complex or the first frame after tricuspid valve closure) and end‐systole (the frame just before tricuspid valve opening or the point when the ventricle appears smallest). All three measurements for each segment were obtained within a single session, and the three values were averaged; the resulting mean was used for subsequent analyses. Myocardial thickness was not measured when the myocardium was invisible (indicated by a visualization score of 0). The observers monitored the echocardiography in real‐time and paused the image when the myocardium was optimally visualized. Measurements were obtained using the caliper tool and then saved and recorded in the picture archiving and communication system.

### 2.4 Visualization

The delineation and myocardial visualization scoring systems were used to evaluate RV wall visualization.

#### 2.4.1 Delineation Scoring

**FIGURE 3:**
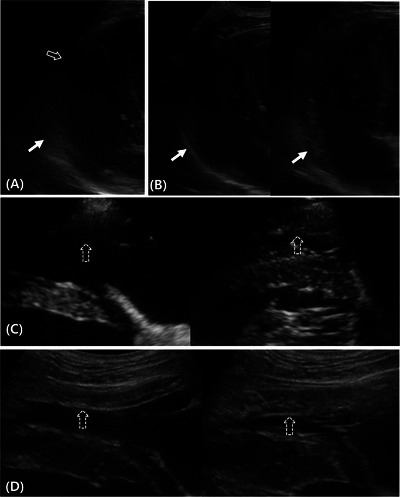
Representative images for myocardial and chamber border delineation score assessments. (A) Right ventricle (RV)‐focused left apical four‐chamber view. Segments B3 (open arrow) and B1 (arrow) show scores of 0 and 1, respectively. (B) RV‐focused left apical four‐chamber view. The images on the left and right show diastole and systole, respectively. Segment B1 (arrows) shows a score of 1. During both systole and diastole, the myocardium is visible. However, the delineation of the endocardial borders remains indistinct. (C) Right parasternal four‐chamber view. Segment A2 (dashed arrows) shows a score of 2. Although segment 2 exhibits indistinct endocardial borders during diastole, clear delineation is observed during systole. (D) Right parasternal four‐chamber view. The left and right images show diastole and systole, respectively. Segment A2 (dashed arrows) shows a score of 3, indicating that the endocardial border is clearly visible during diastole and systole.

This scoring system was designed to evaluate myocardial and chamber border clarity using the sector transducer with B‐mode (ST‐B mode), convex transducer with B‐mode (CT‐B mode), and convex transducer with contrast‐enhanced mode (CT‐CE mode). The scores range from 0 to 3, with 0, 1, 2, and 3 indicating no visible myocardium, a visible myocardium but unclear endocardial borders, visible endocardial borders during the systole or diastole, and clear endocardial border visualization during the systole and diastole, respectively (Figure 3A –D).

#### 2.4.2 Myocardial Visualization Scoring

**FIGURE 4:**
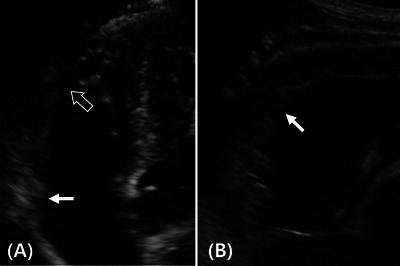
Representative images of myocardial visibility score assessments. (A) Right ventricle‐focused left apical four‐chamber view. Segment B3 (open arrow) shows a score of 1, which appears uniformly hypoechoic because of a rib shadowing artifact. Segment B1 (arrow) shows a score of 2, indicating focal hyperechoic blurring that partially limits comprehensive myocardial assessment, although myocardial heterogeneity remains observable. (B) Left cranial transverse heart base view. Segment C2 (arrow) shows a score of 3, allowing the detailed evaluation of myocardial heterogeneity and texture.

This scoring system was designed to evaluate the quality of myocardial visualization using the ST‐B and CT‐B modes. Based on a four‐point scale, score 0 indicates no visible myocardium; score 1 indicates visible myocardium appearing uniformly hypoechoic because of artifacts, such as rib shadowing, lung interference due to gas‐related reverberation, or tissue‐related factors (fat, muscle, and fibrosis); score 2 indicates partial myocardial heterogeneity and texture visibility because of some artifacts or unclear areas; and score 3 indicates minimal artifacts with clear myocardial heterogeneity and texture visualization (Figure 4A,B).

### 2.5 Contrast‐Enhanced Echocardiography

In this study, SonoVue (Bracco, Milan, Italy), a second‐generation contrast agent made of sulfur hexafluoride‐filled microbubbles, was used. For a single‐view evaluation, 0.2 mL of the ultrasound contrast agent was administered, followed by a flush using 5 mL of normal saline (0.9% NaCl). Contrast agent administration was performed as a rapid bolus in a single motion, followed by saline flushing in approximately 2 s, with an effort to maintain a consistent flush rate across all injections. The bolus of contrast agent was infused through a 24‐ga catheter placed in the cephalic vein, with a washout period of 6 min between injections. After each injection, ultrasound videos were recorded for 2 min and stored digitally. Each contrast agent vial was entirely used within 2 h of opening. Peak pixel intensity, defined as the maximum pixel value within the region of interest, was obtained from the scanner's on‐console readout and reported on an 8‐bit gray scale (0–255; unitless).

### 2.6 RV Fractional Area Shortening

Following the guidelines of the American Society of Echocardiography, the European Association of Cardiovascular Imaging, and consensus from recent veterinary reports, the size and function of the RV were assessed using an RV‐focused view [26, 27]. This involved positioning the transducer one intercostal space cranial to the standard left apical four‐chamber view and adjusting the angle to maximize the RV's longitudinal dimension. Specifically, the aim was to capture the largest RV basal diameter, the longest RV long axis, and the entire RV free wall, while visualizing the left atrium and ventricle. The transducer was positioned to exclude the left ventricular outflow tract to prevent RV foreshortening on echocardiography [1, 28].

**FIGURE 5:**
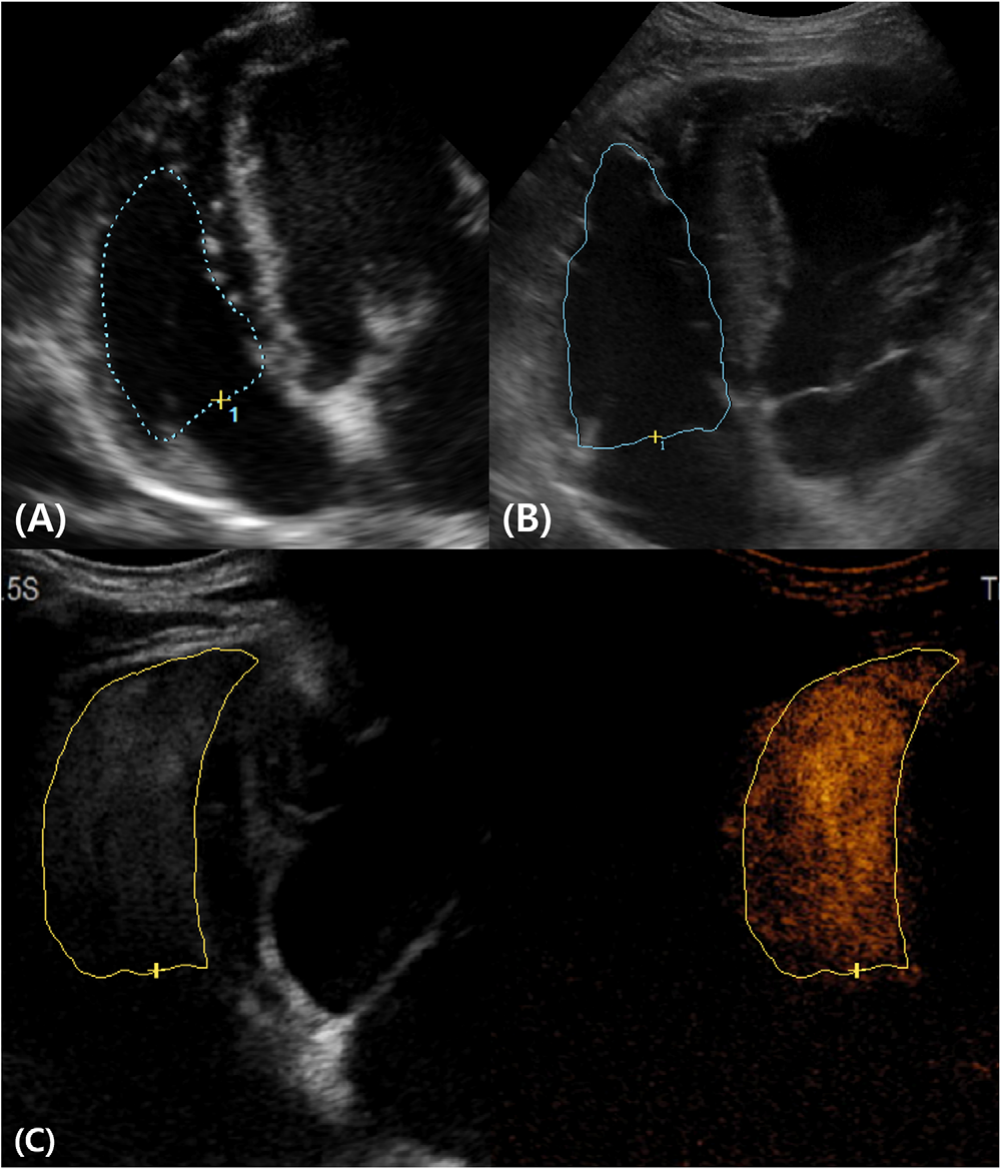
During fractional area change measurement, the end‐diastolic area was assessed using (A) the ST‐B mode; (B) the CT‐B mode; and (C) the CT‐CE modes. The apex was better visualized using the CT‐CE mode, resulting in a larger measured area. CT‐B mode, convex transducer with B‐mode; CT‐CE mode, convex transducer with contrast‐enhanced mode; ST‐B mode, sector transducer with B‐mode.

The areas used for RVFAC calculation were obtained by delineating the RV's endocardial border at end‐diastole and end‐systole (Figure 5A–C). The percentage of RVFAC was then determined as follows:

FAC = (end diastolic area − end systolic area) ÷ end diastolic area × 100

### 2.7 Statistical Analyses

Statistical analyses were performed using SPSS version 29.0 (IBM Corp., Armonk, NY, USA). The statistical differences between myocardial thickness measurements obtained using the convex and sector transducers were compared using a paired sample *t*‐test. For myocardial thickness measurements, observer reliability was evaluated using Cronbach's alpha to assess the intraclass correlation coefficient (ICC). Analysis of variance was used to evaluate myocardial visualization, followed by Scheffé’s post hoc test and the Kruskal–Wallis test for multiple comparisons. *p* value < 0.05 indicated statistically significant differences. The statistical tests were selected in consultation with a statistician, and all analyses were performed by the authors.

## 3 Results

### 3.1 Myocardial Thickness

In total, 2076 myocardial thickness measurements were obtained from 10 beagles. Table 1 summarizes the means and standard deviations of each segment's thickness at end‐diastole and end‐systole. In all views, segment thickness did not differ significantly between the ST‐B and CT‐B modes (*p *> 0.05). In the LCTHB view, based on measurement in the CT‐B mode, segment C2 had the lowest mean thickness (2.5 mm). In the right ventricle‐focused left apical four‐chamber (RV‐LAFC) view, based on measurement in the ST‐B mode, segment B1 had the highest mean thickness (4.5 mm).

**TABLE 1:** Measurement of myocardial segment thickness at end‐diastole and end‐systole.

View	Segment (*n* = 2076)	Phase	Transducer	Thickness (mm)
RPFC	A1	Diastole	CT‐B mode	3.50.39
RPFC	A1	Diastole	ST‐B mode	3.50.51
RPFC	A1	Systole	CT‐B mode	5.30.63
RPFC	A1	Systole	ST‐B mode	5.20.69
RPFC	A2	Diastole	CT‐B mode	3.40.24
RPFC	A2	Diastole	ST‐B mode	3.30.34
RPFC	A2	Systole	CT‐B mode	5.50.54
RPFC	A2	Systole	ST‐B mode	5.30.52
RPFC	A3	Diastole	CT‐B mode	3.20.38
RPFC	A3	Diastole	ST‐B mode	30.36
RPFC	A3	Systole	CT‐B mode	50.62
RPFC	A3	Systole	ST‐B mode	4.90.61
RV‐LAFC	B1	Diastole	CT‐B mode	4.30.5
RV‐LAFC	B1	Diastole	ST‐B mode	4.50.62
RV‐LAFC	B1	Systole	CT‐B mode	5.80.78
RV‐LAFC	B1	Systole	ST‐B mode	6.10.69
RV‐LAFC	B2	Diastole	CT‐B mode	3.60.28
RV‐LAFC	B2	Diastole	ST‐B mode	3.70.38
RV‐LAFC	B2	Systole	CT‐B mode	5.10.56
RV‐LAFC	B2	Systole	ST‐B mode	5.40.53
RV‐LAFC	B3	Diastole	CT‐B mode	3.30.45
RV‐LAFC	B3	Diastole	ST‐B mode	3.50.56
RV‐LAFC	B3	Systole	CT‐B mode	4.60.66
RV‐LAFC	B3	Systole	ST‐B mode	4.70.54
LCTHB	C1	Diastole	CT‐B mode	40.84
LCTHB	C1	Diastole	ST‐B mode	4.30.6
LCTHB	C1	Systole	CT‐B mode	6.20.81
LCTHB	C1	Systole	ST‐B mode	6.30.95
LCTHB	C2	Diastole	CT‐B mode	2.50.38
LCTHB	C2	Diastole	ST‐B mode	2.70.39
LCTHB	C2	Systole	CT‐B mode	5.20.58
LCTHB	C2	Systole	ST‐B mode	50.57
LCTHB	C3	Diastole	CT‐B mode	30.38
LCTHB	C3	Diastole	ST‐B mode	3.20.48
LCTHB	C3	Systole	CT‐B mode	4.50.61
LCTHB	C3	Systole	ST‐B mode	4.80.8

*Note*: Thickness data are presented as means and standard deviations.

Abbreviations: CT‐B mode, convex transducer with B‐mode; LCTHB, left cranial transverse heart base view; RPFC, right parasternal four‐chamber view; RV‐LAFC, right ventricle‐focused left apical four‐chamber view; ST‐B mode, sector transducer with B‐mode.

ICCs were calculated to evaluate the agreement between the two observers’ myocardial thickness measurements. In the RPFC view, segment A2 and A3 thickness measurements had a higher inter‐observer agreement when the CT‐B mode was used than when the ST‐B mode was used. Indeed, the thickness measurement of segment A2 at end‐systole using the CT‐B mode had the highest ICC value (0.92). In the RV‐LAFC view, the thickness measurement ICCs for all segments were comparable between the CT‐B and ST‐B modes, with segment B3 showing low inter‐observer agreement for both transducers. In the LCTHB view, segment C2 and C3 thickness measurements at end‐systole in the CT‐B mode exhibited a higher inter‐observer agreement than when the ST‐B mode was used (Table 2).

**TABLE 2:** Myocardial segment thickness intraclass correlation coefficients (ICCs).

View	Segment (*n* = 2076)	Phase	Transducer	ICC
RPFC	A1	Diastole	CT‐B mode	0.7
RPFC	A1	Diastole	ST‐B mode	0.73
RPFC	A1	Systole	CT‐B mode	0.41
RPFC	A1	Systole	ST‐B mode	0.6
RPFC	A2	Diastole	CT‐B mode	0.55
RPFC	A2	Diastole	ST‐B mode	0.3
RPFC	A2	Systole	CT‐B mode	0.92
RPFC	A2	Systole	ST‐B mode	0.54
RPFC	A3	Diastole	CT‐B mode	0.35
RPFC	A3	Diastole	ST‐B mode	0.12
RPFC	A3	Systole	CT‐B mode	0.85
RPFC	A3	Systole	ST‐B mode	0.69
RV‐LAFC	B1	Diastole	CT‐B mode	0.7
RV‐LAFC	B1	Diastole	ST‐B mode	0.75
RV‐LAFC	B1	Systole	CT‐B mode	0.73
RV‐LAFC	B1	Systole	ST‐B mode	0.65
RV‐LAFC	B2	Diastole	CT‐B mode	0.65
RV‐LAFC	B2	Diastole	ST‐B mode	0.59
RV‐LAFC	B2	Systole	CT‐B mode	0.76
RV‐LAFC	B2	Systole	ST‐B mode	0.6
RV‐LAFC	B3	Diastole	CT‐B mode	0.5
RV‐LAFC	B3	Diastole	ST‐B mode	0.38
RV‐LAFC	B3	Systole	CT‐B mode	0.48
RV‐LAFC	B3	Systole	ST‐B mode	0.4
LCTHB	C1	Diastole	CT‐B mode	0.5
LCTHB	C1	Diastole	ST‐B mode	0.5
LCTHB	C1	Systole	CT‐B mode	0.37
LCTHB	C1	Systole	ST‐B mode	0.37
LCTHB	C2	Diastole	CT‐B mode	0.5
LCTHB	C2	Diastole	ST‐B mode	0.46
LCTHB	C2	Systole	CT‐B mode	0.91
LCTHB	C2	Systole	ST‐B mode	0.26
LCTHB	C3	Diastole	CT‐B mode	0.12
LCTHB	C3	Diastole	ST‐B mode	0.06
LCTHB	C3	Systole	CT‐B mode	0.8
LCTHB	C3	Systole	ST‐B mode	0.44

Abbreviations: CT‐B mode, convex transducer with B‐mode; LCTHB, left cranial transverse heart base view; RPFC, right parasternal four‐chamber view; RV‐LAFC, right ventricle‐focused left apical four‐chamber view; ST‐B mode, sector transducer with B‐mode.

### 3.2 Delineation Scoring

A total of 240 visualization scores were available for analysis. Table 3 summarizes the visualization scores for the delineation between the myocardium and the RV chamber using ST‐B, CT‐B, and CT‐CE modes.

**TABLE 3:** Myocardium‐chamber delineation visualization scores.

View	Segment (*n* = 360)	Transducer	Score	*K*	*p* value	Scheffé
RPFC	A1	CT‐CE mode	2.950.16	11.639	0.003*	*A*
RPFC	A1	CT‐B mode	2.70.42	11.639	0.003*	*A*
RPFC	A1	ST‐B mode	2.40.32	11.639	0.003*	*B*
RPFC	A2	CT‐CE mode	2.80.35	1.674	0.433	
RPFC	A2	CT‐B mode	2.90.32	1.674	0.433	
RPFC	A2	ST‐B mode	2.80.26	1.674	0.433	
RPFC	A3	CT‐CE mode	1.950.64	8.817	0.012*	*B*
RPFC	A3	CT‐B mode	2.70.35	8.817	0.012*	*A*
RPFC	A3	ST‐B mode	2.20.59	8.817	0.012*	*A*
RV‐LAFC	B1	CT‐CE mode	1.40.46	16.631	<0.001***	*B*
RV‐LAFC	B1	CT‐B mode	2.50.41	16.631	<0.001***	*A*
RV‐LAFC	B1	ST‐B mode	2.350.47	16.631	<0.001***	*A*
RV‐LAFC	B2	CT‐CE mode	1.80.54	0.792	0.673	
RV‐LAFC	B2	CT‐B mode	20.76	0.792	0.673	
RV‐LAFC	B2	ST‐B mode	1.90.62	0.792	0.673	
RV‐LAFC	B3	CT‐CE mode	2.450.64	7.423	0.024*	*A*
RV‐LAFC	B3	CT‐B mode	1.550.64	7.423	0.024*	*B*
RV‐LAFC	B3	ST‐B mode	1.70.53	7.423	0.024*	*B*
RV‐LAFC	B4	CT‐CE mode	2.750.49	6.922	0.031*	*A*
RV‐LAFC	B4	CT‐B mode	2.20.58	6.922	0.031*	*B*
RV‐LAFC	B4	ST‐B mode	2.20.41	6.922	0.031*	*B*
RV‐LAFC	B5	CT‐CE mode	2.80.54	5.468	0.065	
RV‐LAFC	B5	CT‐B mode	2.90.34	5.468	0.065	
RV‐LAFC	B5	ST‐B mode	2.50.5	5.468	0.065	
RV‐LAFC	B6	CT‐CE mode	2.60.57	1.395	0.498	
RV‐LAFC	B6	CT‐B mode	2.90.32	1.395	0.498	
RV‐LAFC	B6	ST‐B mode	2.90.42	1.395	0.498	
LCTHB	C1	CT‐CE mode	2.60.47	1.414	0.493	
LCTHB	C1	CT‐B mode	2.40.46	1.414	0.493	
LCTHB	C1	ST‐B mode	2.50.643	1.414	0.493	
LCTHB	C2	CT‐CE mode	2.60.47	2.986	0.225	
LCTHB	C2	CT‐B mode	2.90.46	2.986	0.225	
LCTHB	C2	ST‐B mode	2.70.48	2.986	0.225	
LCTHB	C3	CT‐CE mode	2.30.59	1.466	0.480	
LCTHB	C3	CT‐B mode	2.450.64	1.466	0.480	
LCTHB	C3	ST‐B mode	20.88	1.466	0.480	

*Note*: Score data are presented as means and standard deviations. Scheffé’s post hoc test: *A* > *B*.

Abbreviations: CT‐B mode, convex transducer with B‐mode; CT‐CE mode, convex transducer with contrast‐enhanced mode; *K*, Kruskal–Wallis test; LCTHB, left cranial transverse heart base view; RPFC, right parasternal four‐chamber view; RV‐LAFC, right ventricle‐focused left apical four‐chamber view; ST‐B mode, sector transducer with B‐mode.

***, **, and * indicate *p *< 0.001, <0.01, and <0.05, respectively.

With all transducers, the visualization scores were relatively higher in the RPFC view than in the RV‐LAFC and LCTHB views. In segment A2, the visualization scores were markedly high, with all transducers scoring >2.8 without statistically significant differences between their scores. In segment A3, the CT‐B mode had higher visualization scores when compared with the ST‐B and CT‐CE modes. In the RV‐LAFC view, the visualization scores of apical segments B3 and B4 were significantly higher when the CT‐CE mode was used than when using the CT‐B and ST‐B modes. However, in the LCTHB view, the transducers’ visualization scores did not differ significantly.

### 3.3 Myocardial Visualization Scoring

In myocardial visibility assessment, when compared to the sector transducer, using a convex transducer in the RPFC view significantly improved visibility in all segments (*p* < 0.001). In the RV‐LAFC view, the visualization scores across all segments did not differ significantly. In the LCTHB view, the segment C2 visualization scores of the convex and sector transducers had a significant difference, with the convex transducer achieving higher scores (*p* < 0.05). However, for segments C1 and C2, the scores of the two transducers were not significantly different. Table 4 summarizes the myocardial visibility visualization scores. In all dogs, even in the absence of lesions, such as cardiac tumors, the myocardium exhibited variable thickness and heterogeneous echogenicity. With the use of contrast agents, the myocardium exhibited a uniform mild enhancement, with a peak intensity (the maximum pixel intensity level within the region of interest) of approximately 30 (8‐bit gray scale, 0–255; unitless).

**TABLE 4:** Myocardial visibility visualization scores.

View	Segment (*n* = 240)	Transducer	Score	*p* value
RPFC	A1	CT‐B mode	2.30.48	<0.001***
RPFC	A1	ST‐B mode	1.40.52	<0.001***
RPFC	A2	CT‐B mode	2.90.32	<0.001***
RPFC	A2	ST‐B mode	1.80.63	<0.001***
RPFC	A3	CT‐B mode	2.20.42	<0.001***
RPFC	A3	ST‐B mode	1.10.57	<0.001***
RV‐LAFC	B1	CT‐B mode	1.91.3	1
RV‐LAFC	B1	ST‐B mode	1.90.57	1
RV‐LAFC	B2	CT‐B mode	1.20.92	0.324
RV‐LAFC	B2	ST‐B mode	1.60.84	0.324
RV‐LAFC	B3	CT‐B mode	0.60.7	0.408
RV‐LAFC	B3	ST‐B mode	0.90.88	0.408
RV‐LAFC	B4	CT‐B mode	20.67	0.264
RV‐LAFC	B4	ST‐B mode	2.30.48	0.264
RV‐LAFC	B5	CT‐B mode	2.50.7	0.264
RV‐LAFC	B5	ST‐B mode	2.80.42	0.264
RV‐LAFC	B6	CT‐B mode	2.20.63	0.5
RV‐LAFC	B6	ST‐B mode	20.67	0.5
LCTHB	C1	CT‐B mode	1.70.95	0.458
LCTHB	C1	ST‐B mode	20.82	0.458
LCTHB	C2	CT‐B mode	2.80.63	0.002**
LCTHB	C2	ST‐B mode	1.80.63	0.002**
LCTHB	C3	CT‐B mode	2.10.7	0.109
LCTHB	C3	ST‐B mode	1.50.85	0.109

*Note*: Score data are presented as means and standard deviations.

Abbreviations: CT‐B mode, convex transducer with B‐mode; LCTHB, left cranial transverse heart base view; RPFC, right parasternal four‐chamber view; RV‐LAFC, right ventricle‐focused left apical four‐chamber view; ST‐B mode, sector transducer with B‐mode.

***, **, and * indicate *p *< 0.001, <0.01, and <0.05, respectively.

### 3.4 Fractional Area Change

Two independent observers obtained 60 measurements from 10 dogs. The average fractional area change measured using the CT‐CE mode (36.1%) was significantly lower when compared with the ST‐B and CT‐B modes (48% and 47.5%, respectively). However, the CT‐CE mode's ICC (0.7) was higher, with the ST‐B and CT‐B modes showing lower reliability (Table 5).

**TABLE 5:** Comparison of right ventricular fractional area change (RVFAC) measurements and the intraclass coefficient values obtained using different transducers.

Transducer	RVFAC (%) (*n* = 60)	*K*	*p* value	Scheffé	ICC
ST‐B mode	486.9	27.286	<0.001***	*A*	0.5
CT‐B mode	47.56.5	27.286	<0.001***	*A*	0.47
CT‐CE mode	36.17.3	27.286	<0.001***	*B*	0.7

*Note*: RVFAC data are presented as means and standard deviations. Scheffé’s post hoc test: *A* > *B*.

Abbreviations: CT‐B mode, convex transducer with B‐mode; CT‐CE mode, convex transducer with contrast‐enhanced mode; ICC, intraclass correlation coefficient; *K*, Kruskal–Wallis test; ST‐B mode, sector transducer with B‐mode.

***, **, and * indicate *p *< 0.001, <0.01, and <0.05, respectively.

## 4 Discussion

Accurate evaluation of the RV is essential in veterinary cardiology but remains difficult because of its crescentic geometry, separated inflow/outflow tracts, prominent trabeculations, and the susceptibility of functional indices to preload and afterload [1, 2, 3]. These anatomical and physiological factors hinder quantitative assessment, and poor endocardial delineation can further compromise the accuracy of parameters such as RVFAC. Moreover, RV evaluation is understudied, particularly in dogs, which has led to the reliance on qualitative assessment in clinical practice [1, 29]. In this study, we compared a conventional sector transducer, a convex transducer, and a convex transducer with a contrast agent to comprehensively evaluate right ventricular parameters. This approach enabled the clear visualization and precise measurement of these key factors and contributed valuable insights to the existing literature about right ventricular assessment in veterinary medicine. This study's findings offer guidance on the preferred RV evaluation methods for dogs.

Here, myocardial thickness was measured in three views, and mean values were calculated. The myocardial thickness obtained in this study was smaller than previously reported reference values [29]. Here, the use of different views and measurement methods limited direct comparability with the previous study [6]. Although the previous study used the M‐mode method to measure myocardial thickness in the right parasternal short‐axis view, we used three different views as previously described and obtained the thickness through simple caliper measurements in the B‐mode. Additionally, the previous study used data from various dog breeds with a wide range of body weights, derived a functional relationship between body weight and myocardial thickness, and provided estimated myocardial thickness mean values. Because the previous study focused on larger dog breeds, with an average weight of 26.1 kg, the functional relationship might not apply to small dogs with lower body weights. Here, we exclusively examined beagle dogs (weight: 8–11 kg), which may account for the differences in the results. To establish more comprehensive myocardial thickness reference values, future studies with larger sample sizes should involve more dog breeds and body weights. A previous study reported that in dogs weighing 9 kg, the diastolic and systolic RV parietal wall thicknesses were 5.5 and 6.2 mm, respectively. Although the beagles in this study also had an average body weight of about 9 kg, there were no cases with the reported average diastolic thickness of 5.5 mm.

The delineation scoring system revealed that segment A2 (mid RV free wall) had consistently high scores across all transducers, probably because of its central and near‐field location. In contrast, with the CT‐B mode, segments A1 (basal RV free wall) and A3 (apical RV free wall) exhibited significantly improved visualization scores than with the ST‐B mode. This highlights the advantage of convex transducers in near‐field structure visualization through a wider acoustic window and higher resolution [30]. These characteristics are likely to have enhanced the visualization of the side‐located segments A1 and A3. Therefore, along with the conventional sector transducer, additional assessment with a convex transducer is recommended for the precise evaluation of myocardial segments located in the near field, such as in myocardial tumor cases. In the RV‐LAFC view, compared with the CT‐B and ST‐B modes, the CT‐CE mode significantly improved apical segment (B3, B4) visualization, which is consistent with human studies, where contrast agents enhanced four‐chamber apex visualization. However, because of acoustic shadowing, the basal segments (B1, B6) showed no improvement, which offset the benefits of contrast agents [23]. Therefore, contrast agents are particularly valuable for RV apex assessment or detecting apical lesions, such as thrombi.

The myocardial visualization scoring system evaluated myocardial echogenicity and texture visibility using the CT‐B and ST‐B modes. Because the myocardium has intrinsic structural variability at the macroscopic and cellular levels, it appears heterogeneous on echocardiography, even in the absence of pathological lesions. The myocardium is made of multiple layers (subendocardium, myocardium, and epicardium) and diverse cell types, including endothelial cells, smooth muscle cells, and leukocytes, which contribute to its heterogeneity [31]. Therefore, when interpreting echocardiographic findings, these unique variations should be considered. In the RPFC view, across all segments, the CT‐B mode demonstrated significant improvements when compared with the ST‐B mode. In the LCTHB view, for segment C2, which is located in the mid RV free wall in the near field, the CT‐B mode showed notable improvements when compared with the ST‐B mode. This is attributed to the convex transducer's better spatial resolution, which highlights its utility in near‐field structure evaluation [30].

To evaluate measurement repeatability, including inter‐operator variability, a previous RV myocardial thickness study calculated variation and repeatability coefficients using a random effect model [23]. The previous study reported a repeatability coefficient RC value of 1.7, indicating excellent repeatability. The present study calculated ICC values for all myocardial thickness measurements, which varied across different segments. It is therefore important to be careful when measuring the thickness of segments with low ICC values. Segments with high ICC values offer reliable myocardial thickness mean values, which provide valuable clinical practice and future research insights. Our findings indicate that in all segments of the RPFC view, using the CT‐B mode resulted in significantly higher myocardial thickness measurement ICC values when compared to the ST‐B mode, with segment A2 exhibiting excellent reproducibility. Similarly, in the LCTHB view, in segments C2 and C3, end‐systolic myocardial thickness ICC values were higher with the CT‐B mode than the ST‐B mode, with segment C2 showing an excellent ICC value (0.91). These findings suggest that using a convex transducer enables a more reliable assessment of near‐field lesions, such as suspected RV tumor invasion and cardiomyopathy, which highlights its potential for clinical application. In the RV‐LAFC view, with both transducers, segment B3 exhibited a low inter‐observer agreement, probably because of visualization challenges. Because contrast agents have been shown to enhance myocardial‐chamber boundary delineation, future studies should investigate their use to improve myocardial thickness measurement accuracy.

Here, RVFAC measurement and evaluation of its ICC revealed that RVFAC values significantly decreased with contrast agent use. The normal range of RVFAC values for beagles using sector transducers has been reported to vary with body weight [1]. Considering the beagles in our study weighed 7–12 kg, the normal range based on the previous study is 36.4%–63.7%, which is relatively broad [1]. In our study, the RVFAC values obtained with the contrast agent were slightly lower than this reference range, probably because the RV area assessment was affected by the apex region being measured as larger (Figure 5C). Furthermore, the highest ICC values were observed with the use of a contrast agent, probably because it improved apical visualization and enhanced measurement accuracy. This suggests that contrast‐enhanced echocardiography may be a valuable tool for RV function assessment, which warrants further validation through comparison with cardiac MRI.

A previous study was limited because it used the M‐mode from a single view [29]. This study focused on utilizing perspectives that enable a more effective RV structure evaluation by using the RPFC, RV‐LAFC, and LCTHB views. The RPFC and RV‐LAFC views provide comprehensive RV visualization, from the base to the apex, whereas the LCTHB view is useful for RV base assessment [2]. This comprehensive approach allows for a more detailed and precise RV evaluation.

Convex and linear transducers are typically used in contrast‐enhanced ultrasound [32]. Linear transducers, due to their high frequency and superior spatial resolution, are particularly recommended for detailed imaging of small and superficial structures. In contrast, convex transducers, with their lower frequency and greater penetration depth, are more suitable for evaluating deeper organs [32]. Considering the heart's complex anatomy, convex transducers may offer advantages in evaluating the intricate internal structures in contrast‐enhanced ultrasound examinations. Here, in healthy dogs, contrast echocardiography revealed homogeneous and mild contrast enhancement in the myocardium. In humans, myocardial tumors are reported to exhibit strong contrast enhancement [33]. Therefore, contrast‐enhanced echocardiography is expected to be a useful tool for the evaluation of lesions, such as tumors.

Various SonoVue doses have been used in previous reports [19, 23, 34]. In this study, we administered a 0.2 mL bolus followed by a flush with 5 mL normal saline (0.9% NaCl), which is consistent with the recommended pediatric dose when adjusted for dog weight and has been used effectively in previous echocardiographic studies [23, 35, 36]. Here, using the 0.2 mL dose facilitated myocardial assessment and provided clear myocardial‐chamber delineation. Using higher doses might cause excessive acoustic shadowing, making evaluation more challenging. Additionally, a lower dose can expedite contrast agent clearance, resulting in a shorter washout time. This approach minimized discomfort and adverse effects, with all dogs receiving three injections per evaluation without complications.

The RV chamber fills rapidly, immediately after the contrast agent bolus injection, with strong contrast signals peaking within 5–10 s. Initially, the high‐intensity signals cause attenuation in the basal and medial segments, limiting a clear visualization of the myocardial wall because of incomplete chamber opacification. As the attenuation subsides, the RV becomes fully opacified, followed by myocardial enhancement [23]. However, if too much time passes after the injection, myocardial and atrial enhancement become similar, making it hard to accurately assess the myocardial wall. Therefore, the optimal myocardial wall evaluation window was determined to be 3–6 cardiac cycles postinjection, when chamber opacification was complete, and the myocardial wall delineation was most distinct. Therefore, in this study, the myocardium‐chamber delineation assessment and FAC measurement were done in this window.

A previous study reported a positive correlation between heart rate and FAC [1]. However, in this study, the heart rate could not be controlled, which may have introduced variability across subjects. The heart rates ranged from 62 to 140 beats per minute (bpm), with most heart rates falling between 84 and 127 bpm. Although heart rates varied by up to 30 bpm, most subjects exhibited a heart rate variation of about 10 bpm, indicating relatively consistent heart rates during echocardiographic measurements. However, because previous studies suggest that significant deviations from the normal heart rate can be clinically important, caution should be taken when applying these findings in clinical practice [1].

This study is limited by a small sample size, which may have reduced the statistical power and generalizability of its findings and potentially limited our ability to detect significant differences between groups. Future studies with larger sample sizes are needed to validate these results. Additionally, the experimenters’ awareness of ultrasound contrast agent use may have introduced a bias toward perceived myocardial visualization improvement. Our study is also limited by the use of only one ultrasound device, as other equipment might have obtained different results. Furthermore, right ventricular segmentation was based on the observers’ subjective judgment. Although predefined criteria were established to maintain consistency across views, some degree of subjectivity may have influenced the evaluation and interpretation of myocardial and endocardial borders.

In conclusion, this study demonstrated that for the evaluation of right heart structures, including the RV wall and interventricular septum, the convex transducer allows for more reliable myocardial thickness measurements and improved visualization in near‐field regions compared with the sector transducer. These findings provide valuable right ventricular thickness foundational data and highlight the potential utility of the convex transducer in diagnosing and monitoring heart diseases in dogs, where precision is critical. Furthermore, contrast agent use markedly improved apical visualization in the RV‐LAFC view, resulting in more reliable RVFAC measurements. Although these findings require validation through comparison with MRI‐obtained RVFAC values, they suggest that using convex transducers and contrast agents may enable a more precise assessment of myocardial functional states. Finally, in dogs, the very mild and uniform contrast enhancement exhibited by the normal myocardium is expected to serve as valuable baseline data for diagnosing conditions like cardiac tumors.

## Author Contributions

**Yeonju Park**: conceptualization, methodology, formal analysis, investigation, visualization, writing – original draft. **Suyeon Yoon**: investigation, data curation. **Sumin Han**: investigation, data curation. **Jihye Shin**: investigation, data curation. **Minhyung Kim**: investigation, data curation. **Seungjo Park**: supervision, investigation, writing – review and editing.

## Disclosure

An abstract of this study was presented at the 23rd Federation of Asian Veterinary Associations Congress, Daejeon on October 25, 2024, and it was published in the conference proceedings.

## Ethics Statement

All procedures involving animals were reported in compliance with the ARRIVE 2.0 guidelines.

## Conflicts of Interest

The authors declare no conflicts of interest.

## Data Availability

The data that support the findings of this study are available from the corresponding author upon reasonable request.
